# Development of the continuous ambulatory vestibular assessment (CAVA) system to provide an automatic diagnosis for vestibular conditions: protocol for a multicentre, single-arm, non-randomised clinical trial

**DOI:** 10.1136/bmjopen-2024-085931

**Published:** 2024-11-07

**Authors:** John S Phillips, Stephen J Cox, Gregory Howard, Juliet High, Louisa Murdin, Ian Nunney, Peter Rea, Lee Shepstone

**Affiliations:** 1 University of East Anglia Faculty of Medicine and Health Sciences, Norwich, UK; 2 School of Computing Sciences, University of East Anglia, Norwich, UK; 3 Norwich Clinical Trials Unit, University of East Anglia Faculty of Medicine and Health Sciences, Norwich, UK; 4 Guy’s and Saint Thomas’ NHS Foundation Trust, London, UK; 5 Norwich Medical School, University of East Anglia Faculty of Medicine and Health Sciences, Norwich, UK; 6 Departments of Neuroscience and Informatics, University of Leicester, Leicester, UK; 7 University of East Anglia, Norwich, UK

**Keywords:** Otolaryngology, Audiology, Neurotology

## Abstract

**Introduction:**

Dizziness is a common symptom that can occur in an unpredictable and episodic manner leading to the imprecise reporting of symptoms. Patients will often see many specialists before receiving a diagnosis and treatments can vary in terms of risk and invasiveness which places a significant burden on health services. Achieving an early precise diagnosis could be key in reducing the impact of symptoms on patients and health services.

**Methods and analysis:**

The continuous ambulatory vestibular assessment (CAVA) trial is a single-arm, non-randomised, multicentre diagnostic accuracy device trial that aims to quantify the extent to which the CAVA system can differentiate three common inner-ear causes of dizziness: Ménière’s disease, vestibular migraine and benign paroxysmal positional vertigo. 85 participants with an established diagnosis from each of the three dizziness conditions, a total of 255 participants, will be recruited from ear, nose and throat, audiology and audiovestibular medicine departments in National Health Service (NHS) sites across the UK. The CAVA device is composed of two components: A set of bespoke single-use sensor arrays that adhere to the left and right side of the participant’s face; and a small reusable module fitting over the ear that contains a battery, a data storage facility and connection ports for the arrays. The CAVA device will be worn by all participants for up to 30 days with the aim of capturing eye movement data during a dizzy attack. The first objective is to develop an algorithm that can discriminate between the three dizziness conditions listed above. The second is to quantify the financial and patient benefits of deployment in the NHS. The final objective is to expedite a plan to deploy the system in the NHS.

**Ethics and dissemination:**

The trial was approved by the West Midlands—South Birmingham Research Ethics Committee and the Medicines and Healthcare products Regulatory Agency (MHRA). REC reference: 22/WM/0229, IRAS Project ID: 317899, and MHRA: CI/2022/0062 /GB. Participants will provide full informed consent and can withdraw for any reason without it affecting their standard care. Dissemination will include publication in peer-reviewed journals, presentations at academic and public conferences including patients and the public and to policymakers and practitioners.

**Trial registration number:**

ISRCTN81218533, trial protocol V.3.1 (25 January 2024).

**Strengths and limitations of this study**

- The continuous ambulatory vestibular assessment (CAVA) system is novel in that it records horizontal and vertical eye movements and head accelerations 24/7 and analysis of these signals when they correspond to dizziness symptoms will allow clinicians to retrospectively evaluate the attacks through review of the data.
- A large number of subjects recruited from centres all over the country will enable the collection of a considerable amount of data for training an artificial intelligence system to diagnose the three conditions listed.
- The device is technically reliable, comfortable to wear and easy to apply and remove.
- The CAVA system relies on the patient experiencing an episode of dizziness while wearing the device.
- The trial is limited to the diagnosis of the three index conditions: Ménière’s disease, vestibular migraine and benign paroxysmal positional vertigo.

## Introduction

 Up to 30% of the general population have experienced dizziness significant enough to interfere with activities at some point over their lifetime.^1^ Dizziness can have many causes and it can often present in an episodic manner and frequently unpredictable, so patients present as ‘normal’ during examination interictally; furthermore, reporting of symptoms is imprecise.^2 3^ Although many balance tests are available, they only provide a snapshot of balance function in the absence of an acute attack.^2^ Patients will see up to five specialists before receiving a diagnosis^4^ and the treatments offered vary in terms of risk and invasiveness. Dizziness places a significant burden on health services.^5^

Dizziness is debilitating. The three most common causes of episodic vertigo include Ménière’s disease (MD), vestibular migraine (VM) and benign paroxysmal positional vertigo (BPPV). MD and VM are conditions which result in unpredictable and violent episodes of extreme rotational vertigo, lasting for several hours. Patients with BPPV experience shorter episodes, provoked by normal movement of the head such as with looking up. Dizzy attacks can cause patients to vomit and will leave them physically exhausted. It can take weeks to recover in full, making everyday activities impossible. Dizziness has a financial impact on patients taking frequent sick leave from work or time off to attend hospital appointments.^6^

Different treatment options are available for the three conditions of interest. Patients with MD may be treated with invasive injections into the middle ear which carry risk of infection, damage to the eardrum and hearing loss. Drugs for VM are not without risk and the process of identifying the correct drug is time-consuming. By contrast, BPPV is treated by a simple head manoeuvre, however, if the manoeuvre is done on a patient with an incorrect diagnosis it is likely to result in repeated and unpleasant symptom provocation without benefit. Delays before receiving the correct diagnosis and treatment prolong the suffering of patients, both in terms of the dizziness itself as well as the uncertainty of their prognosis and when the diagnosis is unclear patients may be offered treatments which are eventually revealed to have been for the incorrect condition. Therefore, there is a benefit in providing quick access to the correct condition-specific treatment.

The relationship between balance and eye movements known as the vestibulo-ocular reflex stabilises images on the retina during head movement. When a malfunction occurs in the pathway involving the ears, eyes and brain, an abnormal eye movement is produced, known as nystagmus.^7^ This is characterised by drifting of the eyes in one direction followed by a corrective rapid flick of the eyes in the opposite direction. Different diseases produce different patterns of nystagmus, for example, predominantly horizontal (peripheral vestibular nystagmus), predominantly torsional (BPPV), predominantly vertical (causes outside the peripheral vestibular system).^8^,^10^ The ability to characterise nystagmus will allow us to differentiate between the various vestibular causes of a ‘dizzy attack’ and in the future may provide insights into mechanisms underlying other disorders.

CAVA (continuous ambulatory vestibular assessment) is a wearable device developed to detect nystagmus. As some dizziness can be provoked by the movement of the head,^11^ the CAVA device also records the movement of the head in three axes. The eye movement data captured by the CAVA device is a critical parameter for assessing patients with dizziness.

The CAVA system was evaluated during a clinical investigation involving 17 healthy volunteers. Algorithms were developed to detect physiologically induced nystagmus and achieved an extremely high degree of sensitivity and specificity.^12^ The device was technically reliable, patients were happy to wear it and it was safe.^13^ Participants found the device to be easy to apply and remove and found it to be comfortable to wear. They had little difficulty sleeping while wearing the device and reported that it did not interfere greatly with their daily activities. They did not feel self-conscious wearing the device. From the initial proof-of-concept prototypes, the CAVA device was designed and confirmed to record maximum ocular excursions and eye movements as small as 5 degrees. These tests took place on several subjects with different head shapes, ensuring that recording was not limited by physiological differences or by placement on the face.

Previous approaches to record vertigo attacks include: (1) Wolf described a portable device for recording two channels of eye movement to a floppy disk for 10 minutes.^14^ This system did not allow for continuous monitoring and is now technologically obsolete. (2) Rauch proposed an ambulatory device for recording physiological parameters including ECG, respiration rate, skin temperature, head and eye movement.^15^—this device is no longer in development. (3) Young used video cameras mounted in swimming goggles to record the eyes but patients close their eyes during attacks and attacks can start during sleep.^16^ Wolf’s and Young’s systems are applied at the onset of vertigo which is challenging for attacks that begin without warning. The CAVA system overcomes these limitations and has been proven to be effective in previous research.

During our most recent clinical investigation (NCT04026516), the device recorded the first-ever full example of an acute attack of MD revealing a novel period of nystagmus preceding a full attack.^17^ The data from the CAVA device has allowed a greater insight into the symptoms reported by individuals with an otherwise identical history.^18^ We have also confirmed that a variety of nystagmus signals exhibiting different characteristics can be identified reliably from the data recorded for which we developed novel artificial intelligence (AI) techniques.^12 17 19^

We have completed proof-of-concept work showing that the CAVA system provides information to allow our target diseases to be differentiated by way of five parameters: Nystagmus duration, whether the nystagmus changes direction, the slow phase velocities (speed of the drifting motion), the nystagmus slow phase duration and whether the nystagmus is motion-provoked.^20^ These findings are supported by Young *et al*.^16^ The next step is to demonstrate the consistency of these findings among a larger group of patients to develop algorithms to automatically discriminate these conditions and to determine their diagnostic accuracy.

### Aims and objectives

We will develop the CAVA system’s capabilities from detecting nystagmus to diagnosis based on the novel, long-term data it records. This work is supported by our proof-of-concept data showing that our objectives are feasible. We will demonstrate the potential economic and patient benefits offered by the device and instigate a plan to deploy the system into the National Health Service (NHS).

Aim: Establish to what extent the data from the CAVA system can be used to differentiate three of the most common inner-ear causes of dizziness.

Primary Objective: Enable the CAVA system to differentiate between MD, VM and BPPV.

Objective 2: Quantify the health and economic benefits of an alternative diagnostic pathway involving the CAVA system.

Objective 3: Finalise and instigate a project-exit plan to deploy the CAVA system into general clinical use in the UK publicly funded healthcare systems, the NHS and internationally.

## Methods and analysis

### Trial design

This is a single-arm, non-randomised multicentre diagnostic accuracy study using a medical device including an assessment of health economics and the formation of a commercialisation plan.

### Study setting

Participant recruitment will be from ear, nose and throat (ENT) departments, audiology departments and departments of audiovestibular medicine in NHS institutions distributed across sites in the UK ensuring a broad diversity of participants in terms of gender, age, socioeconomic group and ethnicity.

### Patient and public involvement

Three volunteers with dizziness conditions have been involved in early testing of the device, development of trial materials and are included as members of our trial management groups (TMGs) and steering committee to advise on study progress and dissemination. A separate patient and public involvement event with individuals with dizziness conditions was held to gain feedback on the acceptability of the device and trial processes.

### Population

We will seek patients with a confirmed diagnosis of one of the three conditions of interest (unilateral MD,^21^ VM,^22^ unilateral posterior canal BPPV^23^ currently experiencing symptoms within 4 weeks prior to recruitment.

Inclusion criteria:

1. Age 18 and over.
2. Must have relevant index medical condition: Meniere’s: MD, VM, posterior canal BPPV.
3. Experiencing episodes of true vertigo with at least two episodes within the preceding 4 weeks at the time of consent.
4. The vertigo is of a duration and a nature supportive of the relevant index medical condition.
5. It is advisable that participants have access to a phone so they can contact the research team with any issues.
6. Willing to provide informed consent.
7. Willing to comply with the study protocol for using the CAVA device.
8. Willing to complete all study materials.
9. Adequate grasp of the English language or language used within an existing translated version of the informed consent form and patient information sheet and where hospital translators are available to provide support.

Exclusion criteria:

1. Has an allergy to plasters and/or medical adhesives.
2. Evidence of dermatitis, fragile skin or any other condition that could be aggravated by the repeated application of skin surface adhesives.
3. Pregnant or breastfeeding mothers.
4. Bilateral or second side MD.
5. Active bilateral or second-side posterior canal BPPV.
6. Currently enrolled on an intervention trial (not including questionnaire-based or observational trial).
7. Patients who meet diagnostic criteria for more than one eligible condition at the time of recruitment.

### Recruitment and screening

255 participants, 85 per target disease, will be recruited from ENT departments, audiology departments and departments of audiovestibular medicine through NHS hospitals. Up to a total of 400 participants may be recruited in order that up to 30 days of data (and/or at least one qualifying dizziness attack with nystagmus) from the device is collected from 255 individuals.

Potential participants will be identified by staff familiar with the trial and the relevant departments in NHS institutions in the UK. Posters will also be used to inform participants of the trial.

Potential participants will be provided with study information either in person or by post/email. They may also be contacted by phone or they may contact the clinical research team directly. All potential participants will be offered the opportunity to discuss the advantages and disadvantages of study participation with a member of the research team and/or their physician before providing consent. See online supplemental materials for a copy of the participant consent form.

### Blinding

Blinding is not possible due to the design of the trial. Participant diagnosis is confirmed as part of the inclusion criteria, there are no separate arms of the trial and all participants will wear the device in the same manner. The primary outcome is objectively assessed.

### Intervention

All participants fulfilling the eligibility criteria and providing consent will wear the CAVA device for up to 30 days. To balance the consequences of delaying BPPV treatment with not being able to identify the nystagmus associated with BPPV, individuals with BPPV will be offered a particle positioning manoeuvre after 4 days of wear. The CAVA algorithm will then diagnose each patient based only on the data recorded from the device. The accuracy of the system will be tested by comparing the CAVA system’s diagnosis with each patient’s known diagnosis. The results of the algorithm work (‘the diagnosis’) will not be available until the end of the trial and will not be made available to clinicians or patients during the trial.

### The CAVA device

The CAVA system will eventually consist of two separate elements: Hardware and software. The software will be developed from the data collected from this trial and is not fully developed or under test in this trial.

**Figure 1:**
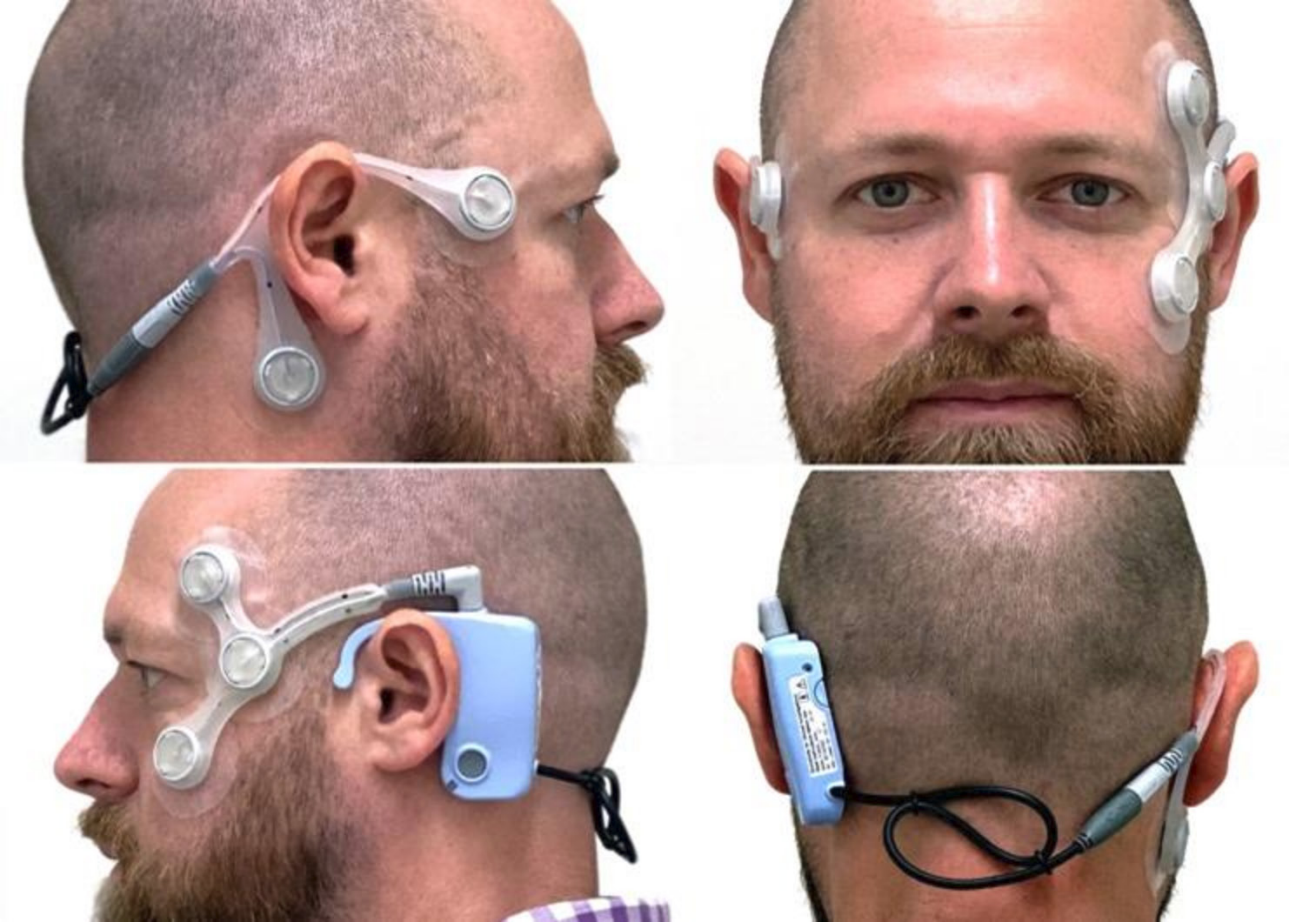
A doctor modelling the CAVA device. Consent for publication of this image was received. CAVA, continuous ambulatory vestibular assessment.

- The hardware is composed of two parts. First, there is a reusable device that sits over the ear and records eye and head movements; this is comprised of a logging module containing a battery, microcontroller, accelerometer, data storage facility, an event marker button and connection ports. Second, there are two single-use sensory arrays (electrodes) that adhere to the participant’s face (figure 1).
- The software will consist of a computer algorithm which will compute head and eye data recorded during a 30-day period. The data used to undertake this work will be uploaded onto a computer from an SD card stored in the device. This data analysis is performed ‘off-line’ after the participant has worn the device.

The device manufacturer is Wright Design Limited. A series of preliminary prototype devices have been manufactured and tested as part of a preliminary programme of work aimed at developing the overall project to this stage. This work has been completed by the primary research team in collaboration with a design agency (Wright Design Limited). The device is a lightweight, durable, body-worn monitoring device that can be worn day and night. It will be worn on the face and head but will minimally intrude into the patient’s normal lifestyle.

The electrode array comprises two separate electrode mounts containing five adhesive electrodes in total. These semiflexible cables are linked by a detachable connector to the logging unit which rests behind the left ear. The mounts are designed to be replaced by the subject on a daily basis. The electrodes are biocompatible to skin for the required duration of wear, this is in order to maximise user tolerance of wearing the device and to reduce the possibility of skin irritation.

The device also includes an indicator LED which glows either green to indicate normal device operation or otherwise shows a flashing/stationary green/red light pattern. Data captured by the device is stored on an inbuilt SD card. This data is intended to be downloaded and viewed by the trial computing team. Data can be downloaded by way of a USB-B interface. Neither the SD card nor the USB-B interface are accessible to the participant wearing the device, minimising the chance of damage to the device or loss of data.

**Figure 2:**
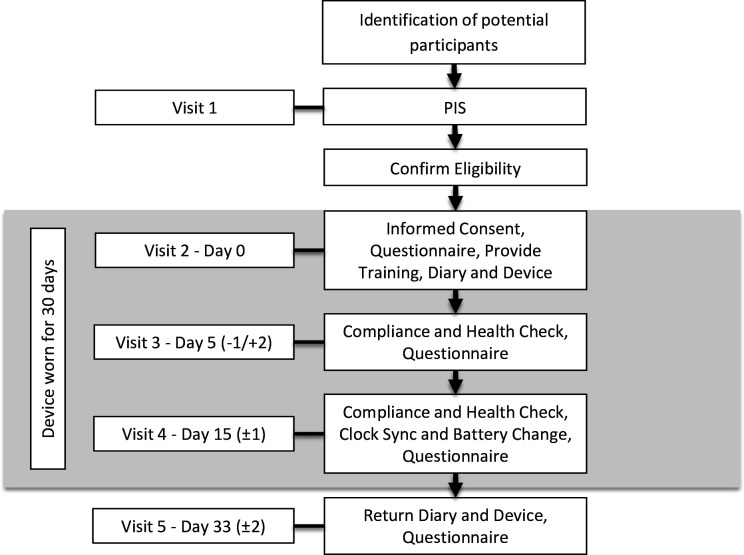
Participant timeline. PIS, Patient Information Sheet

The device is powered by a single AAA battery lasting a minimum of 18 days which is the maximum duration between hospital visits. Site staff will be trained in changing the battery and this will take place on visit 4 (figure 2). Participants do not need to change the battery or open the device in any way.

The device will commence logging once the logging unit has been initialised on a computer by a member of the research team which includes the installation of a battery into the device. The event marker button can be depressed to check the status of the device. If the device is functioning properly and connected to the face, the status LED will glow green. Data captured from the device is stored in a text format with each day stored in a separate date-identifiable file.

The electrodes used with the device are intended to be worn for a 23-hour period and all of the equipment should be removed and stored by the participant until the equipment can be returned to the clinic. Once the device and electrode array have been removed, participants can shower or wash before applying a new electrode array to their face as described in the instructions provided to them. They will repeat this process daily. At the end of 30 days, they will return the device to the clinic. The device will be shipped back to the trial team where experts will download and analyse the data from the device. For downloading, the device will be connected to a computer by USB and will appear as a mass storage device on the computer. Files can then be removed from the device as would be normal for any USB storage device. During this file transfer procedure, the device will be powered by USB rather than the internal battery.

The CAVA device is non-sterile and is designed and manufactured under a trial quality management system developed in line with EN13485.

Preliminary prototype devices have been developed to be able to record the maximal ocular excursions and velocities that are observed in clinical practice. These preliminary prototype devices have consistently, accurately, precisely and reliably identified periods of physiological nystagmus in subjects both stationary and in motion and have produced consistent signals at the beginning and end of each trial period.

CAVA is a class 1 medical device worn externally for the purpose of recording data relating to a non-vital physiological process. As such, CAVA poses extremely low levels of risk to participant safety and no risk is posed in the event of failure of the device’s primary function. Device malfunctions will be reported as part of normal trial processes and will only be subject to expedited reporting if the malfunction could have led to an adverse event.

CAVA is currently a prototype device and is not currently intended to assist with clinical diagnoses or treatment. Although the overall function of the device will not change, its appearance as depicted in this document may be subject to minor alterations throughout the device’s development.

### Outcomes

#### Primary outcome

The primary outcome will be nystagmus identification using the algorithm. This will be derived for each participant by comparing the actual nystagmus data and the device-derived nystagmus data. Data will be collated to calculate an overall value for sensitivity and specificity for all participants.

#### Secondary outcomes

Secondary outcomes include safety events, device compliance, health economics, commercialisation, average time to first dizzy attack, patient acceptability and device malfunctions.

Device compliance will be calculated for each participant by appraising the percentage of time the device was worn for each day of the trial as well as for the entire 30-day trial. Data will be collated to calculate an overall value for device compliance for all participants.

The health economic study will inform the market access plan which will be produced after the study detailing any of CAVA’s financial and other patient benefits to inform the route to its commercialisation. This will be developed using study outputs as well as data from consultation with stakeholders regarding the feasibility of adoption within the NHS, patient and public (PPI) meetings and public dissemination events.

Patient acceptability will be assessed through the use of a bespoke questionnaire, completed by participants at the end of their time in the trial.

Device malfunctions will be recorded, both participant-reported (and perceived) and those noted by the clinical or data analysis teams.

Safety events (adverse events) will be reported by participants and recorded throughout the trial.

### Sample size

A minimum sample size of 85 participants for each of the three conditions is required to demonstrate a minimum diagnostic sensitivity of 95% and a specificity of 95% with a 3% margin of error for each condition.

### Retention

The trial intervention period is relatively short, so retention is not expected to be too challenging. It is anticipated that some participants will not wear the CAVA device or have a dizziness attack while wearing the device within 30 days, so the sample size is inflated to allow for this. Some non-conformance will be apparent or self-reported by the participant, others will only be detected once the data is downloaded at the end of the 30 days. Therefore, recruitment will continue until the device data analysis team confirms 255 participants with one qualifying dizziness event or the maximum upper approved limit of 400 participants is reached.

A potential challenge with retention is participants who drop out not returning the CAVA device. As this could impact the rest of the trial, participants are offered a £20 voucher incentive on return of the device and electrodes.

### Data analysis

The device data will be downloaded by the investigator and securely backed up locally as well as uploaded to the University of East Anglia’s secure research storage for permanent (tape backup) storage. The software will be used to convert the data from its encoded form on the SD card into the waveform samples provided by the device. The signals will then be resegmented into files, each file containing a full calendar day.

To train the algorithms, we require a collection of nystagmus waveform segments collected from a patient each labelled with the underlying diagnosis of the patient’s condition. The nystagmus waveforms from a patient’s data will be gathered by the computational team in two ways. First, by finding the positions of presses of the event marker in the signal and searching around these points for nystagmus-like signals; second by reading the patient’s diary entries and searching for signals at times when they have noted spells of dizziness (of course, these two conditions may often co-occur). Signals in these regions will be carefully assessed for possible evidence of nystagmus and any candidate waveforms will be copied and stored. Any relevant comments by the patient will be copied and stored with the accompanying waveforms. We will also use automatic analysis. We have already developed algorithms that can detect nystagmus-like waveforms with high accuracy and these will be used to both verify any waveforms found by the procedure above and to discover any other nystagmus events that the patient was either unaware of or had not marked.

After confirmation of nystagmus waveforms associated with the three target conditions of the study, algorithms will be built and tested to do automatic diagnosis. The most suitable computational technique for this task is machine learning (ML), sometimes known as ‘AI’. When using ML to make classifications, we aim to make maximum use of the available labelled data without falling into the trap of testing on material that is either part of the training set or is correlated with it which could lead to optimistically biased results. Hence the paradigm used here is to train on all the data except for that from a single subject and then test on that subject’s data. This procedure is repeated for each subject in turn and the average error rate and its SD are estimated. This technique is known as cross-validation.

An ML algorithm learns how to adjust automatically its parameters in such a way that it will make measurements on the waveforms that maximise the classification accuracy on the three diseases. Although it would not be obvious what these measurements are, it is possible to analyse the final ML model in such a way that we can later understand what the important criteria for distinguishing between the three diseases are. However, this process requires adequate data. If there is not enough data for the algorithm to learn the correct measurements, we can ‘help’ it by providing it with automatic measurements of features that we think may be important such as slow and fast phase velocities and amplitudes and measured nystagmus frequencies. We are in the process of incorporating these parameters.

Automatic diagnosis tests will be run periodically throughout the active stage of the project to identify potential problems in the data and to check that diagnosis accuracy continues to improve as more data becomes available. The final reported results will represent the diagnostic success rate of the project. The data should prove to be a rich source for further analysis that may considerably increase our understanding of the three target conditions and related matters.

A data validation plan will be developed to describe the downloading, storage and validation of the data in more detail and a statistical analysis plan will be finalised before analysis of the data.

### Economic evaluation

The health economic plan is to ascertain the NHS, personal and intangible costs involved in previously diagnosed patients versus what they might have required had they worn the device for a period of 1 month’s monitoring. We shall develop an early cost-effectiveness model that will describe both these pathways using the data collected in the study. The effectiveness measure is likely to be obtaining a definitive diagnosis but we shall also allude to quality of life with the disease as a secondary measure of effectiveness when appropriate. We expect the model to be robust using standard probabilistic sensitivity analysis and by obtaining resource use standardised units, values using standard NHS and other unit costs (PSSRU, 2020). We will also collect impacts on personal costs such as out-of-pocket costs, lost wages and income from time-off work and changes to longer-term productivity using standard average UK wage rates.

The economic study will also collect quality of life data using the EQ-5D (3-level) questionnaire to ascertain the short-term and longer-term impact of episodes of dizziness on quality of life. This will inform the design of future clinical trials as well as the preliminary economic model and play an important role in informing whether quality of life is impacted by the different methods of diagnosis.

Data will be collected at each point of patient contact during the monitoring period, collecting data on the resources used and any productivity losses by patients in the preceding days/weeks. Quality of life data using the EQ-5D will be collected at the beginning and at the end of the whole monitoring period. This will help determine the overall quality of life with and without and the frequency of attacks and whether the process of diagnosis itself confers any perceptible changes in quality of life. We shall follow the CHEERS checklist or the Consolidated Health Economic Evaluation Reporting Standards (Husereau, 2022 Drummond M *et al*) in our work.

### Commercialisation

A commercialisation consultant will oversee a portfolio of work to support the commercialisation of the CAVA system. They will produce a market access plan detailing CAVA’s route to commercialisation and confirm CAVA’s placement within existing NHS pathways and how it would be implemented. We will build on our previous focus-group work with a stakeholders’ event involving professionals, patients and IT stakeholders from NHS organisations. We will identify the issues important to stakeholders regarding the feasibility of adoption within the NHS and will establish support for adoption. We will host a public event to review the interim findings from the trial and a public dissemination event to share our findings with the public. Throughout the project, we will engage with prospective licensees.

### Data management and monitoring

All data (except data recorded on to the device itself) is collected and managed using REDCap,^24 25^ a secure web-based software platform designed to support data capture for research studies, providing audit trails for tracking data collection and manipulation and automated export procedures to common statistical packages.

Oversight of the trial and data collected is by the TMG and other independent committees, each with their own remit and terms of reference.

CAVA trial team members monitor the trial database and generate reports and review data entry. The essential trial issues, events and outputs, including defined key data points, are discussed by the trial team on a weekly basis and with relevant committees when necessary.

## Ethics and dissemination

This study has been approved by the West Midlands—South Birmingham Research Ethics Committee and the MHRA (REC reference: 22/WM/0229, IRAS Project ID: 317899, MHRA: CI/2022/0062/GB, Clinical Investigation Plan (CIP) V.3.1 dated 25 January 2024). Study sites are included in the application and do not need to obtain separate ethics approval. Participants provide consent to take part and the right of refusal to participant or requests of withdrawal will be respected.

Dissemination plans include publication in peer-reviewed journals, presentations at academic and public conferences consisting of patients and the public and to policymakers and practitioners. The results of the trial will be disseminated regardless of the direction of effect. The statistical analysis will be drafted and approved by the trial’s governance committees prior to data lock. The CAVA systems route to commercialisation will be developed using study outputs as well as data from consultation with stakeholders regarding the feasibility of adoption within the NHS, from PPI meetings and public dissemination events.

### Current study status

The first participant was randomised in February 2023. Recruitment is expected to take 24 months with results expected to be published following the final device return in 2025.

## supplementary material

10.1136/bmjopen-2024-085931 online supplemental file 1
