# Supplementary material for: Development of the continuous ambulatory vestibular assessment (CAVA) system to provide an automatic diagnosis for vestibular conditions: protocol for a multicentre, single-arm, non-randomised clinical trial
Source: BMJ Open. 2024 Nov 7;14(11):e085931. doi: 10.1136/bmjopen-2024-085931 (PMC11552575; doi:10.1136/bmjopen-2024-085931)
Supplement: online supplemental file 1 [file bmjopen-14-11-s001.pdf]

Norfolk and Norwich University Hospitals NHS
NHS Foundation Trust

Informed Consent Form

Continuous Ambulatory Vestibular Assessment (CAVA): Development of a System to Provide an Automatic Diagnosis for Vestibular Conditions

Participant Identification Number:___________ **Please initial box**

1. I confirm that I have read and understand the information sheet dated ………. (version……….) for the above study. I have had the opportunity to consider the information and ask questions. ☐

2. I understand that my participation is voluntary and that I am free to withdraw at any time, without giving any reason, without my medical care or legal rights being affected. ☐

3. <u>If I have BPPV</u>, I understand that my treatment will be delayed, and I understand the implications and risks in delaying the treatment. ☐

4. I understand that the Norfolk and Norwich University Hospitals (NNUH) NHS Foundation Trust is the sponsor for this study based in the United Kingdom. Norwich Clinical Trials Unit is part of the University of East Anglia (UEA), who with the Sponsor, (NNUH), will act as joint Data Controllers for this study. I understand that the University of East Anglia (UEA) will have ownership of all the anonymised trial data from this study. ☐

5. I understand that relevant sections of my medical notes and data collected during the study may be looked at by individuals from my local NHS Trust, the Norwich and Norfolk University Hospitals NHS Foundation Trust, the University of East Anglia and members of the CAVA research team and regulatory authorities where it is relevant to my taking part in this study. I give permission for these individuals to have access to this information in order to cover access to medical records. ☐

6. I understand that I will be asked to complete questionnaires and that quotes from my answers may be anonymised and used in publications. ☐

7. I give permission for my GP to be informed that I am taking part in this study. ☐

8. I understand my data may be kept and used in other research; and only shared after all identifying information was removed and made completely anonymous. ☐

9. I give permission for my contact details and a copy of this consent form to be kept confidentially and securely by the study team and Norwich Clinical Trials Unit ☐

10. Optional: I would like to see the results. If you initial this box please provide your preferred contact method (post or email) to your local NHS team ☐

11. I agree to take part in the above study. ☐

Name of participant	Date	Signature
Name of person taking consent	Date	Signature

Copies: 1 for participant; 1 to be kept with hospital notes; 1 forwarded to NCTU
