# Supplementary material for: Development of the continuous ambulatory vestibular assessment (CAVA) system to provide an automatic diagnosis for vestibular conditions: protocol for a multicentre, single-arm, non-randomised clinical trial
Source: BMJ Open. 2024 Nov 7;14(11):e085931. doi: 10.1136/bmjopen-2024-085931 (PMC11552575; doi:10.1136/bmjopen-2024-085931)
Supplement: Supplementary file 2 [file bmjopen-2024-085931.reviewer_comments.pdf]

PEER REVIEW HISTORY

BMJ Open publishes all reviews undertaken for accepted manuscripts. Reviewers are asked to complete a checklist review form and are provided with free text boxes to elaborate on their assessment. These free text comments are reproduced below.

ARTICLE DETAILS

Title (Provisional)

Development of the Continuous Ambulatory Vestibular Assessment (CAVA) system to provide an automatic diagnosis for vestibular conditions: protocol for a multicentre, single-arm, non-randomised clinical trial

Authors

Phillips, John S; Cox, Stephen J; Howard, Gregory; High, Juliet; Murdin, Louisa; Nunney, Ian; Rea, Peter; Shepstone, Lee

---

VERSION 1 - REVIEW

Reviewer 1

Name Bruintjes, Tjasse D.

Affiliation Leiden University Medical Center, Department of Otorhinolaryngology—Head and Neck Surgery

Date 02-May-2024

COI None

Interesting, well written article on a promising new technique to diagnose episodic dizziness.

I have a few minor remarks.

1. p. 3, line 25: "dizziness can have many causes and its occurence is episodic..". Clearly not all types of dizziness have an episodic character?

2. p. 3, line 43: "MD patients may be treated with highly effective though invasive injections.." This statement doesn't match with the conclusions of two recent Cochrane reviews on IT-treatment (> evidence for use of IT-injection is very uncertain) and should be downsized.

3. p. 5, line 53-54: should the relevant medical condition be unilateral MD (instead of MD) and unilateral p-BPPV (instead of pBPPV)?

4. What happens after the diagnosis pBPPV has been confirmed? Does a patient undergo treatment with a CRM prior to inclusion? If so, the chance of capturing a dizziness attack in the 30 days following inclusion may be minimal. On the other hand, including a patient without performing a CRM seems to be unethical. Please dedicate a few words to this.

**Reviewer**	**2**
**Name**	**Kaski, Diego**
**Affiliation**	**University College London Hospitals NHS Foundation Trust**
**Date**	**06-Jun-2024**
**COI**	**nil**

Thank you for asking me to review this protocol for CAVA: continuous ambulatory vestibular assessment. This is a protocol for a multi-centre diagnostic accuracy (validation) un-blinded trial. I was interested to read this.

I have no major issues with the manuscript. It is clear, well-written, and explains the trial aims and methodology. Some minor comments:

1. The CAVA device records only horizontal and vertical eye movements. How will the authors identify the torsional nystagmus components of some episodes, particularly BPPV, or inferior division vestibular neuronitis?

2. Why are centres limited to ENT and audiovestibular settings? Vestibular migraine, in particular, seems more of a neurological diagnosis. Are Neurology units excluded for a particular reason?

3. Page 4, line 4: "predominantly vertical (outside the vestibular system)". Perhaps this is my ignorance, but is vertical nystagmus not a feature of certain vestibular brainstem or cortical syndromes? As such, this is still vestibular, albeit not peripheral?

4. I note that the CAVA has been given to a set of healthy volunteers. There is also reference to a single patient in whom a Meniere's disease attack was captured. Should there not be a more extensive feasibility/usability study performed, prior to a validation study? Patients with dizziness may have very different views about wearing this device continuously for 30 days, than healthy volunteers.

5. It was not entirely clear to me until the end whether the data from these 255 participants will be used to train the algorithm. If so, there needs to be more information about the power calculations used for this. I was also not clear about what specific features of the nystagmus will be analysed. Are these slow phase velocities?

6. On a related note, what happens if the majority of the individuals do not have typical attacks during the 30-day monitoring period? This may not be unexpected given the nature of some of these conditions (particularly MD and BPPV where attacks might be sporadic and occur across longer inter-ictal timespans).

7. The data analysis seems very labour-intensive at present. Will this be considered as part of the economic analyses? Going forwards, will such a system requiring technician time to analyse the traces?

VERSION 1 - AUTHOR RESPONSE

Reviewer: 1
Prof. Tjasse D. Bruintjes, Leiden University Medical Center, Gelre Hospital
Comments to the Author:
Interesting, well written article on a promising new technique to diagnose episodic dizziness.
I have a few minor remarks.
1. p. 3, line 25: "dizziness can have many causes and its occurence is episodic..". Clearly not all types of dizziness have an episodic character?

We agree with your reviewer's observation and have revised the text to read:

'dizziness can have many causes, and it can often present in an episodic manner' Page 2

2. p. 3, line 43: "MD patients may be treated with highly effective though invasive injections.." This statement doesn't match with the conclusions of two recent Cochrane reviews on IT-treatment (> evidence for use of IT-injection is very uncertain) and should be downsized.

We agree with your reviewer's observation and have revised the manuscript to read: 'MD patients may be treated with invasive injections'. Page 2

3. p. 5, line 53-54: should the relevant medical condition be unilateral MD (instead of MD) and unilateral p-BPPV (instead of pBPPV)?

We agree with your reviewer's observation and have revised the manuscript to read to reflect the terms 'unilateral MD' and 'unilateral p-BPPV'. Page 4

4. What happens after the diagnosis pBPPV has been confirmed? Does a patient undergo treatment with a CRM prior to inclusion? If so, the chance of capturing a dizziness attack in the 30 days following inclusion may be minimal. On the other hand, including a patient without performing a CRM seems to be unethical. Please dedicate a few words to this.

This point was carefully discussed before the trial protocol was agreed by the ethics committee and MHRA. To balance the consequences of delaying BPPV treatment with not being able to identify the nystagmus associated with BPPV, we agreed that for individuals with BPPV, they would be offered a CRM at day four rather than them having to wait the full thirty days. It has been, and is our practice, to fully explain the consequences of delaying treatment, so that participants can make an informed decision regarding whether to take part in this study.

We have revised the manuscript to clarify this point. Page 5

Reviewer: 2
Dr. Diego Kaski, University College London Hospitals NHS Foundation Trust
Comments to the Author:

Thank you for asking me to review this protocol for CAVA: continuous ambulatory vestibular assessment. This is a protocol for a multi-centre diagnostic accuracy (validation) un-blinded trial.

I was interested to read this. I have no major issues with the manuscript. It is clear, well-written, and explains the trial aims and methodology. Some minor comments:

1. The CAVA device records only horizontal and vertical eye movements. How will the authors identify the torsional nystagmus components of some episodes, particularly BPPV, or inferior division vestibular neuronitis?

The nystagmus of BPPV is not purely torsional and this has made it possible for our team to identify torsional nystagmus (Newman JL, Phillips JS, Cox SJ. Reconstructing animated eye movements from electrooculography data to aid the diagnosis of vestibular disorders. Int J Audiol. 2022;61(1):78-83.). We have performed further analyses to finely tune our torsional nystagmus detector. Our refined torsional nystagmus detector is the subject or an oral presentation at the forthcoming American Academy of Otolaryngology meeting in September this year.

2. Why are centres limited to ENT and audiovestibular settings? Vestibular migraine, in particular, seems more of a neurological diagnosis. Are Neurology units excluded for a particular reason?

Neurology units are not excluded, we have just primarily decided to identify Principal Investigators from ENT and Audiovestibular units, because those involved with the development of this research are more familiar with these individuals. The Principal Investigators at participating sites are actively encouraged to work with other teams within their organisation who manage patients with dizziness. Neurologists often see patients with vestibular migraine, Meniere's disease, and BPPV, so they have provided excellent support to date.

3. Page 4, line 4: "predominantly vertical (outside the vestibular system)". Perhaps this is my ignorance, but is vertical nystagmus not a feature of certain vestibular brainstem or cortical syndromes? As such, this is still vestibular, albeit not peripheral?

The text '(outside the vestibular system)' has been changed within revised manuscript to read '(outside the peripheral vestibular system). Page 2

4. I note that the CAVA has been given to a set of healthy volunteers. There is also reference to a single patient in whom a Meniere's disease attack was captured. Should there not be a more extensive feasibility/usability study performed, prior to a validation study? Patients with dizziness may have very different views about wearing this device continuously for 30 days, than healthy volunteers.

The development of the CAVA system: from conception, through development as part of the MRC-funded healthy volunteer trial, as part of the MRC-funded dizziness trial (NCT04026516), and as part of our current NIHR-funded trials, has taken place in partnership with patients and their families. Our current NIHR-funded work includes three patients as part of the research team. One of the patients is a co-applicant on our NIHR award. Our patient advisors have advised us directly, and via facilitated events, regarding a range of matters from suitability of the CAVA device for long-term wear, to how to improve access to wearable diagnostic systems for underserved groups.

5. It was not entirely clear to me until the end whether the data from these 255 participants will be used to train the algorithm. If so, there needs to be more information about the power calculations used for this. I was also not clear about what specific features of the nystagmus will be analysed. Are these slow phase velocities?

Response from Stephen Cox (Computing Lead and Co-Chief Investigator):

The section headed 'Data analysis' has been extended to include a more detailed description of how the data is used in the technique of 'cross-validation' and also to describe how measurements made on the waveforms (such as slow-phase velocities) can be used.

Our medical statistician has determined that a minimum of 85 patients per disease are required to demonstrate the required level of accuracy (95% sensitivity and specificity, with a 3% margin of error). Therefore, we require 255 patients. Page 9

6. On a related note, what happens if the majority of the individuals do not have typical attacks during the 30-day monitoring period? This may not be unexpected given the nature of some of these conditions (particularly MD and BPPV where attacks might be sporadic and occur across longer inter-ictal timespans).

Those individuals not experiencing typical attacks of vertigo will not be included in the final analysis.

7. The data analysis seems very labour-intensive at present. Will this be considered as part of the economic analyses? Going forwards, will such a system requiring technician time to analyse the traces?

Yes, this will form part of our economic analysis.

*** ***

COI statements:

Reviewer: 1
Competing interests of Reviewer: None.

Reviewer: 2
Competing interests of Reviewer: Nil.

VERSION 2 - REVIEW

**Reviewer**	**1**
**Name**	**Bruintjes, Tjasse D.**
**Affiliation**	**Leiden University Medical Center, Department of Otorhinolaryngology—Head and Neck Surgery**
**Date**	**10-Oct-2024**
**COI**	

The comments of the reviewers have been addressed adequately.

I am looking forward to see the results of this study!

Reviewer 2

Name Kaski, Diego

Affiliation University College London Hospitals NHS Foundation Trust

Date 06-Oct-2024

COI

Authors have addressed concerns
